# Evaluating the Antioxidants, Whitening and Antiaging Properties of Rice Protein Hydrolysates

**DOI:** 10.3390/molecules26123605

**Published:** 2021-06-12

**Authors:** Hui-Ju Chen, Fan-Jhen Dai, Cheng-You Chen, Siao-Ling Fan, Ji-Hong Zheng, Yu-Chun Huang, Chi-Fai Chau, Yung-Sheng Lin, Chin-Shuh Chen

**Affiliations:** 1 Department of Food Science and Biotechnology, National Chung Hsing University, Taichung 402204, Taiwan; d103043004@mail.nchu.edu.tw (H.-J.C.); chaucf@nchu.edu.tw (C.-F.C.); 2 Healthmate Co., Ltd., Changhua City 500016, Taiwan; jane@healthmate.com.tw (F.-J.D.); eileen@healthmate.com.tw (S.-L.F.); tina@healthmate.com.tw (Y.-C.H.); 3 Ph.D. Program in Materials and Chemical Engineering, National United University, Miaoli 360001, Taiwan; D0612002@smail.nuu.edu.tw; 4 Department of Chemical Engineering, National United University, Miaoli 360001, Taiwan; U0714049@smail.nuu.edu.tw; 5 Institute of Food Safety and Health Risk Assessment, National Yang-Ming University, Taipei 112304, Taiwan

**Keywords:** rice protein hydrolysate, antioxidant, hyaluronidase, tyrosinase, cosmetic

## Abstract

Plant-derived protein hydrolysates have potential applications in nutrition. Rice protein hydrolysates (RPHs), an excellent source of proteins, have attracted attention for the development of cosmeceuticals. However, few studies have reported the potential application of RPH in analysis, and this study examined their antioxidant activities and the inhibitory activities of skin aging enzymes. The results indicated that the total phenolic and flavonoid concentrations were 2.06 ± 0.13 mg gallic acid equivalent/g RPHs and 25.96 ± 0.52 µg quercetin equivalent/g RPHs, respectively. RPHs demonstrated dose-dependent activity for scavenging free radicals from 1,1-diphenyl-2-picrylhydrazyl [half-maximal inhibitory concentration (IC50) = 42.58 ± 2.1 mg/g RPHs] and 2,2′-azino-bis (3-ethylbenzothiazoline-6-sulfonic acid) (IC50 = 2.11 ± 0.88 mg/g RPHs), dose-dependent reduction capacity (6.95 ± 1.40 mg vitamin C equivalent/g RPHs) and oxygen radical absorbance capacity (473 µmol Trolox equivalent/g RPHs). The concentrations of the RPH solution required to achieve 50% inhibition of hyaluronidase and tyrosinase activities were determined to be 8.91 and 107.6 mg/mL, respectively. This study demonstrated that RPHs have antioxidant, antihyaluronidase, and antityrosinase activities for future cosmetic applications.

## 1. Introduction

Ultraviolet radiation exposure is responsible for photoaging (or extrinsic aging); in contrast, reactive oxygen species produced in cell metabolism and the deterioration of biological functions are responsible for intrinsic aging [1,2]. Processed foods often contain natural antioxidants such as catechins, ascorbic acid, tocopherols, rosmarinic acid, and phenolic extracts from various plants. Research conducted into natural antioxidants now considers nontraditional provenances. Naturally sourced antioxidants are more desirable than chemically produced antioxidants, since some synthetic antioxidants have been reported to be carcinogenic [3]. Rice (*Oryza sativa*) is a major dietary staple for people worldwide, particularly those living in Asia. The globe’s annual rice production is approximately 741 million tons [4]. In Asian countries, rice is reportedly the source of 75% of the energy intake of over 2 billion people [5]. The extensive rice production results in a corresponding amount of by-product production. The residual product from the rice production process contains the majority of the grain’s protein (~60–85%) but is thrown away or used for feeding animals [6,7,8]. Peptides obtained from various protein hydrolysates reportedly act as potential antioxidants [9]. Natural and nontoxic antioxidants can therefore potentially be extracted from food protein hydrolysates. Numerous scholars have employed lipid-rich models and reported protein hydrolysates as well as milk, zein, and soy protein peptides to have crucial antioxidant characteristics, including scavenging of free radicals, inhibition of food and in-vitro lipid peroxidation, and chelation of transition metals [10,11,12].

Hyaluronic acid (HA) helps to rejuvenate the skin because it increases viscosity, contains moisture, and makes extracellular fluids less permeable. Because of its excellent water-holding capacity, HA increases the youthfulness, moisturization, and smoothness of the skin and reduces the degree of wrinkles [13,14]. Unfortunately, the level of HA in the skin naturally decreases with age. Hyaluronidase is an enzyme that destroys HA, causing loss of skin strength, flexibility, and moisture, which in turn, leads to skin aging. Therefore, wrinkles can be treated by inhibiting hyaluronidase and maintaining the HA content of the skin [15,16]. The melanin-producing enzyme tyrosinase contributes vitally to the rate-limiting step of the process through which melanin is produced. Therefore, pigmentation disorders are commonly treated and skin brightening achieved by inhibiting or downregulating tyrosinase activity [17,18].

In several studies, grain protein hydrolysates and the peptides that can be obtained from them have been discovered to have antioxidant, antihypertensive, and antitumor activities [19,20]. The positive contributions to human health of food-originating peptides and proteins are gradually being recognized [21]. Consumers increasingly demand that the cosmetic and health care industries use natural bioactive compounds. Rice protein hydrolysates (RPHs) have attracted attention as an excellent source of proteins. However, few studies have reported the characterization and functional analysis of RPHs. Therefore, this study evaluated the antioxidant activity and hyaluronidase and tyrosinase-inhibitory activities of RPHs.

## 2. Results and Discussion

### 2.1. Total Phenolic Concentration (TPC) and Total Flavonoid Content (TFC)

**Figure 1:**
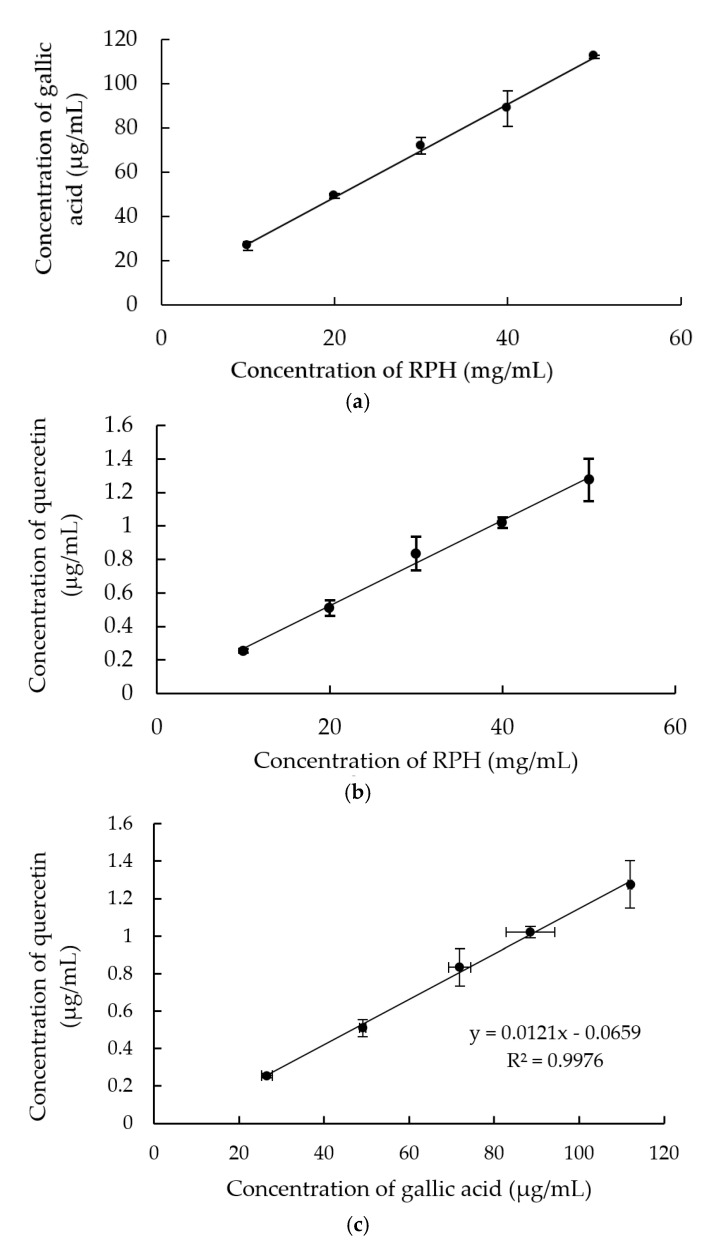
(**a**) Total phenolic concentration (TPC) and (**b**) total flavonoid concentration (TFC) versus rice protein hydrolysate (RPH) concentration. (**c**) relationship between the TPC and TFC of RPH.

The standard in the TPC assay was gallic acid of several concentrations. Higher absorbance indicated a higher TPC. The TPC of the RPH samples was obtained by inputting the RPH samples’ optical absorbance values into the gallic acid calibration curve. By plotting the RPH concentration against the phenolic concentration (Figure 1a), an average TPC of 2.06 ± 0.13 mg GAE/g RPHs was obtained. A TFC of 25.96 ± 0.52 µg QE/g RPHs was obtained by following a similar procedure (Figure 1b). Figure 1c further relates the TPC and TFC of the RPHs samples. It reveals that the relationship between the TPC and TFC and can be expressed as y = 0.0121x + 0.0659, where x and y are the TPC and TFC, respectively.

The standard in the TPC assay was gallic acid of several concentrations. Higher absorbance indicated a higher TPC. The TPC of the RPH samples was obtained by inputting the RPH samples’ optical absorbance values into the gallic acid calibration curve. By plotting the RPH concentration against the phenolic concentration (Figure 1a), an average TPC of 2.06 ± 0.13 mg GAE/g RPHs was obtained. A TFC of 25.96 ± 0.52 µg QE/g RPHs was obtained by following a similar procedure (Figure 1b). Figure 1c further relates the TPC and TFC of the RPHs samples. It reveals that the relationship between the TPC and TFC and can be expressed as y = 0.0121x + 0.0659, where x and y are the TPC and TFC, respectively.

The TPC of RPHs included the concentrations of phenolic amino acids and phenolic compounds of the peptides. Protein–phenolic compound interaction generally involves covalent and noncovalent bonding. Phenolic compounds are released during enzymatic hydrolysis. Specific enzymes may be most able to destroy protein–polyphenol complexes; this results in a greater number of phenolic compounds and peptides with phenolic groups, such as tyrosine, being released [22]. A strong correlation has been reported between the total polyphenol content of grains and their biological activity. Polyphenols are well known to have strong antioxidant activities [23]. Although found in less amount, terpenes [24] or sesquiterpenes [25] in rice could also contribute antioxidant activities.

### 2.2. Activity of Antioxidants

#### 2.2.1. Radical Scavenging Activity of DPPH Free Radicals

**Figure 2:**
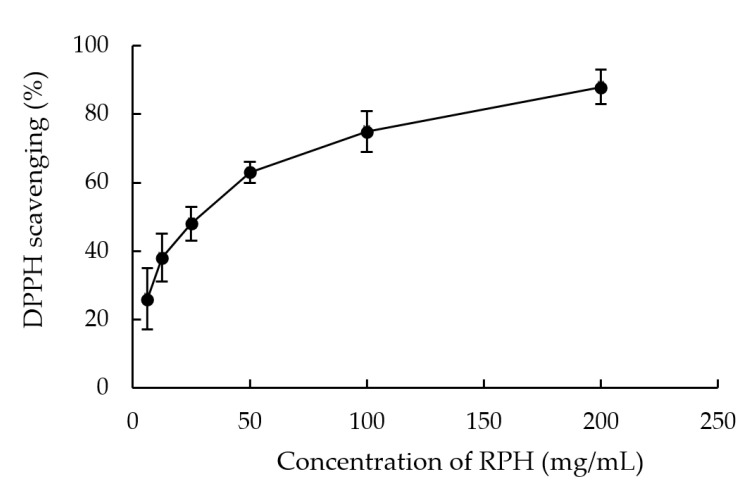
Influence of RPH concentration on the scavenging activity of 1,1-diphenyl-2-picrylhydrazyl (DPPH).

Figure 2 depicts the DPPH free radical scavenging activity in the RPH solution. A higher concentration of the solution was discovered to result in higher activity. The half-maximal inhibitory concentration (IC50), which is the extract concentration for which half of all DPPH free radicals can be scavenged, was 42.58 ± 2.1 mg/mL of rice peptides.

#### 2.2.2. Scavenging Activity of ABTS Free Radicals 

**Figure 3:**
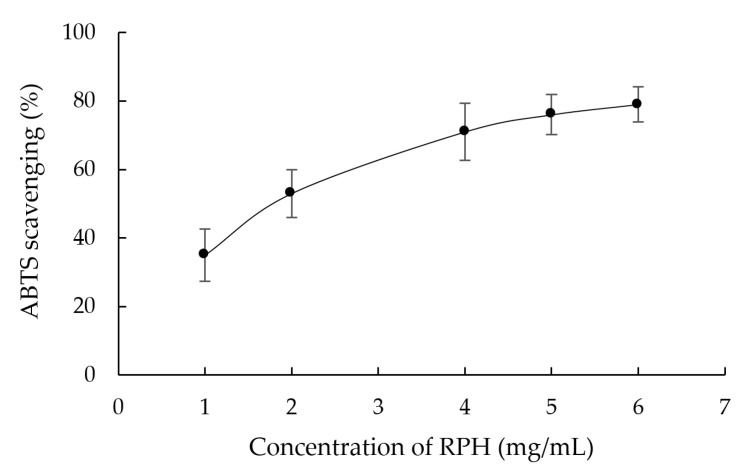
Influence of RPH concentration on the scavenging ability of 2,2′-azino-bis(3-ethylbenzothiazoline-6-sulfonic acid) (ABTS).

RPHs’ ABTS free radical scavenging activity, illustrated in Figure 3, was higher when a greater extract concentration was employed. The IC50 was 2.11 ± 0.88 mg/mL of rice peptides. This result indicated that RPHs had strong ABTS free radical scavenging activity. The sulfur-containing amino acids, including Met and Cys, and hydrophobic amino acids, including Ala, Val, Ile, Leu, Met, Cys, Tyr, Phe, Try and Pro, might be important factors with regard to the ABTS free radical scavenging activity [26]. Moreover, aromatic amino acids, including Phe and Tyr, also promote ABTS free radical scavenging activity because of their benzene rings donating protons to electron deficient radicals [27].

In this study, the IC50 value of ABTS free radical scavenging activity was lower than DPPH free radical scavenging activity, agreeing with results of *Jatropha curcas* L. seed shell and kernel [28] and jujube fruit seed and peel pulp [29]. This finding also corresponds to the report of rice bran protein hydrolysates with 43.98–66.25 µmol Trolox equivalent/g sample and 403.28–430.12 µmol Trolox equivalent/g sample for DPPH free radical scavenging activity and ABTS free radical scavenging activity, respectively [27]. One possible reason is the difference in the solubility between DPPH free radical (oil soluble) and ABTS free radical (oil/water soluble) [30,31]. The antioxidant potential of rice bran protein hydrolysates was influenced by its molecular weight profile, amino acid composition and hydrophobicity [32].

#### 2.2.3. Reduction Capacity

**Figure 4:**
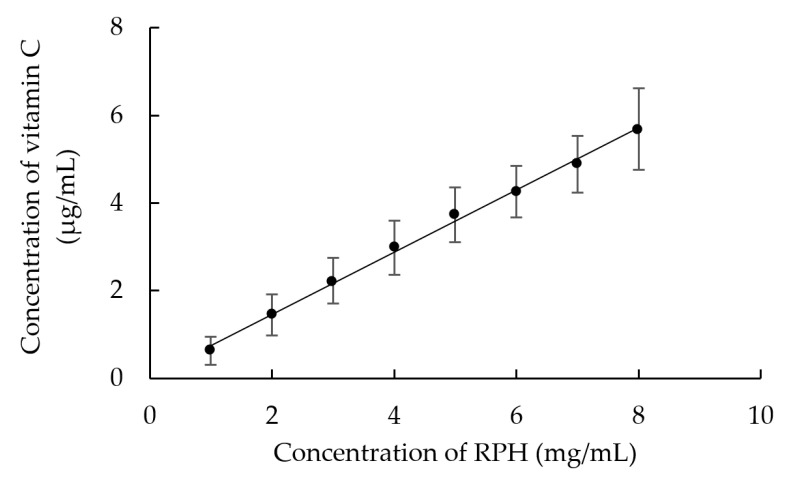
Influence of the concentration of RPH on reduction capacity.

The reduction capacity assay findings for RPHs are presented in Figure 4. The reduction capacity increased with the RPHs concentration. The reduction capacity was 6.95 ± 1.40 mg VCE/g RPHs, indicating that RPHs are an effective antioxidant. 

#### 2.2.4. Oxygen Radical Absorbance Capacity (ORAC)

**Figure 5:**
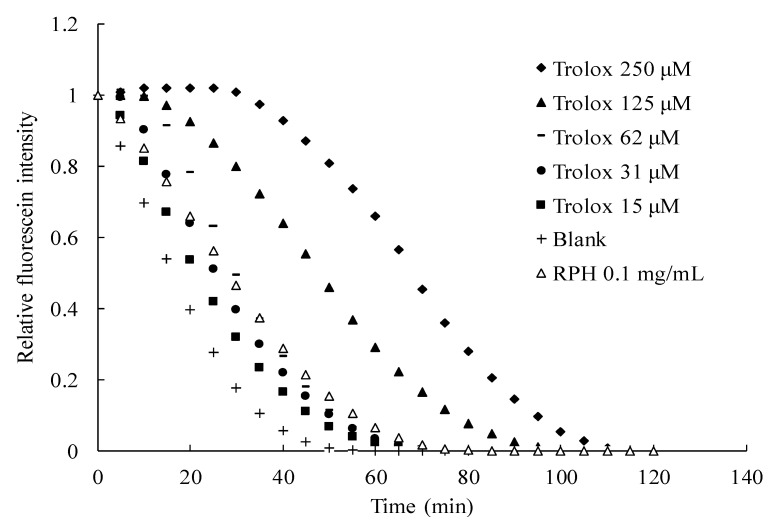
Fluorescence decay kinetic curve of the oxygen radical absorbance capacity assay for various samples.

The ORAC assay has advantages over other approaches to antioxidant activity determination, including the reactants used being peroxy radicals with a similar mechanism of reaction and redox potential to physiological oxidants; the overall charge and protonation state with which the antioxidants react resemble those in the human body [33]. The ORAC method also has biological relevance to the efficacy of antioxidants in the human body. Figure 5 depicts the results of ORAC analysis of RPHs and the Trolox standard in various concentrations. The ORAC was derived from the regression equation of the calibration curve relating the net AUC to the Trolox concentration. The results indicated that RPHs had an ORAC of 473 μmol TE/g RPHs.

Antioxidant peptides or amino acids can be obtained by enzymatic protein hydrolysis, resulting in highly active against oxidants [34]. The metal ion chelation, lipid peroxidation inhibition, and free radical scavenging of biologically active peptides are responsible for their antioxidant activity. Free radicals can be quenched and the expression of oxidative-stress-reducing proteins and enzymes upregulated by antioxidant peptides. The antioxidant efficacy of protein hydrolysates and peptides are reportedly dependent on the sequence of amino acids and size of the peptide, which are affected by the hydrolysis conditions, protein source, and type of protease [35]. According to Adebiyi et al. [36], the largest digestible rice bran protein can be broken into smaller pieces by subtilisin, resulting in greater protein yield and content. A hydrolysate’s TPC and antioxidant activity may be affected by the specificity of enzymes. Therefore, the antioxidant activity of a peptide might be influenced by the protein source’s characteristics, the enzyme’s specificity, and the level of hydrolysis [37].

There are many reports using proteases (such as Alcalase, a commercial name of a subtilisin A from *Bacillus* species) to hydrolyze plant-derived proteins to obtain antioxidant peptides. In this regard, soy protein is one of the most reported proteins [38]. Furthermore, Alcalase hydrolysis of rice bran protein is also found. Under optimal conditions, Alcalase hydrolysis of glutinous rice bran produced protein hydrolysates with the IC50 value of 0.87 ± 0.02 mg/mL in DPPH free radical scavenging [39]. In our study, the IC50 value of RPHs was 42.58 ± 2.1 mg/mL. Although the IC50 value in DPPH free radical scavenging in this study was not as effective as that from rice bran protein, ABTS free radical scavenging (IC50 = 2.11 mg/mL) was more effective than soy protein hydrolysates obtained by Alcalase hydrolysis (IC50 = 2.93 mg/mL) [40].

### 2.3. Hyaluronidase Inhibitory Activity

**Figure 6:**
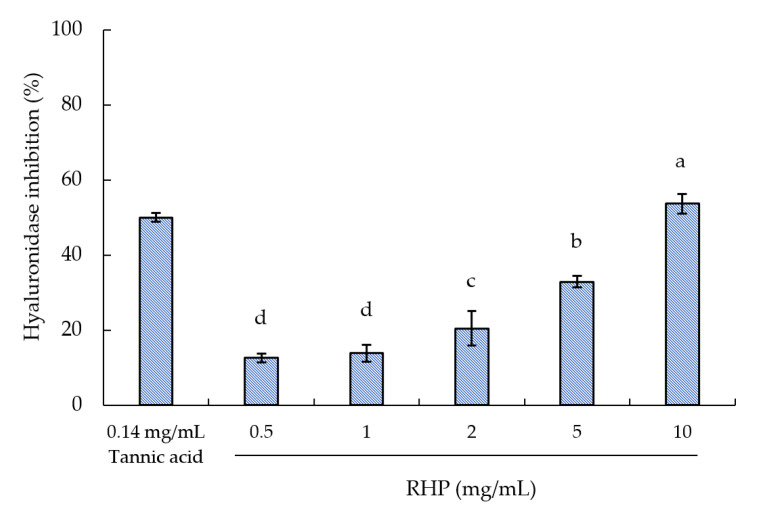
Effect of RPH concentration on hyaluronidase inhibitory activity. The means not sharing a common superscript are significantly different (*p* < 0.05).

A proteolytic enzyme, hyaluronidase, is found in the dermis and catalyzes the degradation of HA in the extracellular matrix [41]. This study employed tannic acid as a positive control for comparison purposes. Figure 6 reveals that tannic acid had a higher hyaluronidase activity inhibition; the IC50 was 0.14 mg/mL, similar to the value obtained by Nishida et al. (0.121 mg/mL; 71.1 mM) [42]. By contrast, an IC50 of 8.91 mg/mL was calculated for the RPH solution. This result of the RPH solution corresponded to our previous IC50 value, 7.61 mg/mL [43]. Proteins, polysaccharides, and plant-originating and synthetic compounds are among the range of compounds in which hyaluronidase inhibitors are present. These inhibitors help maintain the HA synthesis–degradation balance [44]. Low HA concentration in the skin results in dryness and wrinkles. Therefore, inhibition of hyaluronidase activity is a route through which the skin’s morphology can be improved and its aging delayed.

### 2.4. Tyrosinase-Inhibitory Activity

**Figure 7:**
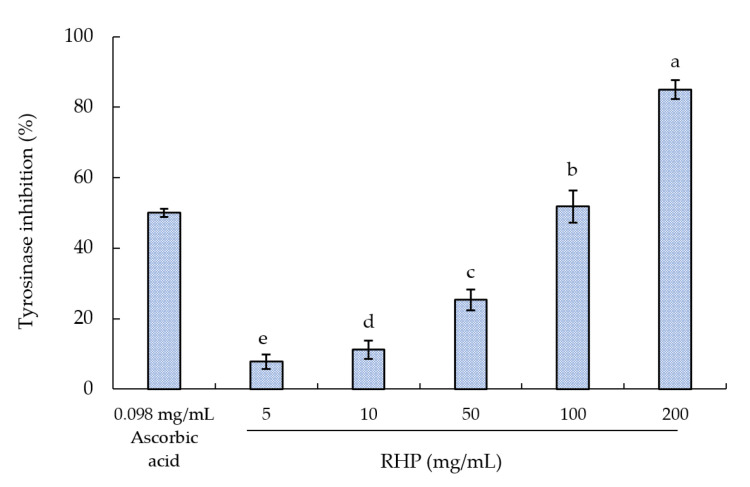
Effect of RPH concentration on tyrosinase-inhibitory activity. Means not sharing a common superscript are significantly different (*p* < 0.05).

Protein hydrolysates from natural sources have the potential to inhibit tyrosinase activity. The in-vitro tyrosinase inhibition test is usually used to evaluate how skin-whitening agents directly affect tyrosinase activity [45]. By participating in the melanin synthesis rate-limiting step, tyrosinase catalyzes L-tyrosine hydroxylation into L-DOPA and then the oxidation of the latter into o-dopaquinone. When it is desirable to prevent biosynthesis of melanin, inhibition of L-tyrosinase activity can be crucial. Here, tyrosinase was used to gage RPH antityrosinase activity. As depicted in Figure 7, the concentration 107.6 mg/mL achieved 50% inhibition of tyrosinase activity. Ascorbic acid exhibited high tyrosinase-inhibitory activity (IC50 = 0.098 mg/mL), which was similar to the 0.102 mg/mL that Seo et al. reported [46]. 

Rice bran protein hydrolysates exhibited significantly high tyrosinase-inhibitory activity [47,48]. The tyrosinase-inhibitory activity of the RPH solution may result from the amino acid profiles of peptides. Schurink et al. described that effective tyrosinase-inhibitory peptides consist of arginine residues and phenylalanine [49]. Tyrosinase-inhibitory activity can be improved by hydrophobic amino acid residues (e.g., alanine), and production of melanin can be disrupted by alanine [50]. Besides, Zhang et al. also reported the rice protein hydrolysate could reduce melanin content and tyrosinase activity in the UVB-induced cell model [51]. 

### 2.5. Amino Acid Profiles and MWs of RPHs 

The rice’s protein content after starch removal in the present study was 23.56% by weight, and the degree of hydrolysis of the sample hydrolyzed by protease was 9.36%. Table 1 details the composition of amino acids in the RPHs. In the sample, each 100 g contained 5.18 g of amino acids. Regarding the amino acid components, RPHs were rich in alanine, leucine, arginine, glutamic acid, and aspartic acid. Each 100 g of the sample contained 1.73 g of hydrophobic amino acids (alanine, valine, leucine, isoleucine, proline, phenylalanine and cysteine) in total. This result was completely different from our previous report [43] in the amylase solution and its treatment temperature to remove starch. The content of hydrophobic amino acids was 1.90 times higher than our previous report. The lower treatment temperature (60 °C) in this study may prevent denaturation of proteins in large quantities, so that the activity of amino acids may be better preserved. In addition, the similar conclusion is also obtained from other fungal amylase and glucoamylase to saccharify starch in white rice bran [52].

Research has found hydrophobic amino acids to resemble antioxidants by raising lipid-based solubility in protein hydrolysates and peptides from various protein sources, thus promoting the interaction with free radicals [38,53]. Some amino acids were reported by Chen et al. [54] to generally be antioxidants; the acids mentioned included tryptophan, cysteine, methionine, tyrosine, and histidine. In the present study, aromatic amino acids (phenylalanine, tyrosine, and tryptophan) comprised 0.53 g/100 g RPHs. Therefore, these peptide-originating amino acids were probably responsible for RPHs’ antioxidant activity.

**Figure 8:**
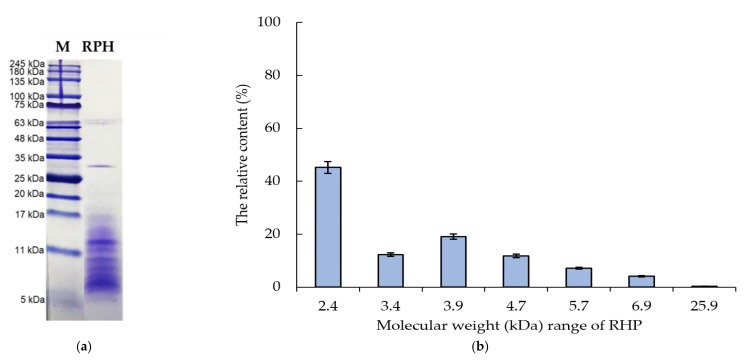
Molecular weight (MW) determination for RPHs through sodium dodecyl sulfate–polyacrylamide gel electrophoresis (SDS-PAGE): (**a**) tricine-SDS-PAGE patterns of the MW marker (line 1) and RPHs (line 2) and (**b**) the relative content (%) of each MW of RPHs.

In addition, proteins that are hydrolyzed into shorter peptides have a different MW distribution, and some hydrophobic groups folded in the interior of the complete natural protein molecules are usually exposed to the aqueous phase. This is related to the protein molecules being structurally rearranged and therefore to the protein’s functional properties [55,56]. The tricine-SDS-PAGE data indicated that the MW of RPHs was in the range 5–35 kDa (Figure 8a).

Figure 8b depicts the relative content of various MWs in RPHs. Overall, 45.24% of all the protein was in the main band (MW ≈ 2.4 kDa). Similar results were obtained regarding the rice bran protein hydrolysates’ peptide. The highest antioxidant activity obtained by Thamnarathip et al. [37] was that for peptides of MW = 6–50 kDa. In addition, relationships exist between protein hydrolysates’ function and the MW distribution and the composition of amino acids [57]. This explains the antioxidant activity of RPHs observed in the present study.

### 2.6. Cell Toxicity Test

**Figure 9:**
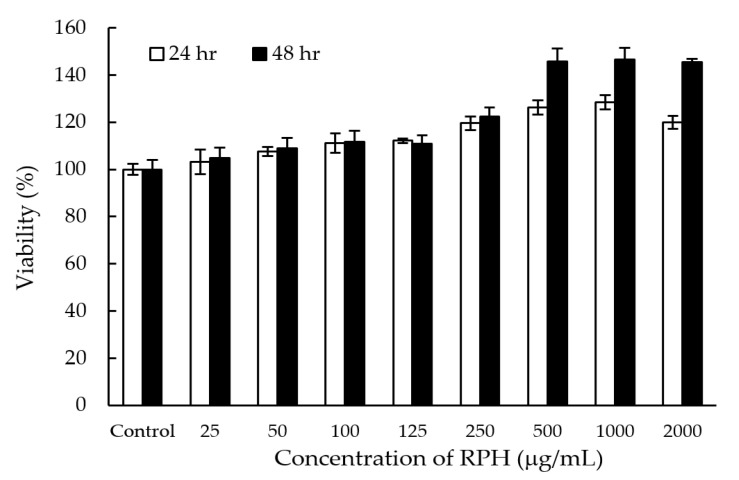
MTT assay for the cell toxicity of raw 264.7 cells treated with different RPH concentrations for 24 and 48 h.

Low cell toxicity is required for future applications. To evaluate the cytotoxicity and biocompatibility of RPHs, the cell viability of raw 264.7 cells in RPH solution was measured via MTT method. As demonstrated in Figure 9, the cell viability was above 100% when treated with 25–2000 µg/mL RPH for 24 h and 48 h. Results indicate the remarkably low cytotoxicity of RPHs. Therefore, RPHs can be potentially used as cosmetic applications with very low cytotoxicity.

## 3. Materials and Methods

### 3.1. Reagents

Iron(III) chloride, 2,2′-azino-bis(3-ethylbenzothiazoline-6-sulfonic acid) (ABTS), Trolox (6-hydroxy-2,5,7,8-tetramethylchroman-2-carboxylic acid), l-3,4-dihydroxyphenylalanine (L-DOPA), 1,1-diphenyl-2-picrylhydrazyl (DPPH), and trichloroacetic acid were acquired from Alfa Aesar (Tewksbury, MA, USA). 2,2′-azobis(2-methylpropionamidine) dihydrochloride (AAPH), Folin–Ciocalteu’s phenol reagent, gallic acid, ascorbic acid, mushroom tyrosinase, and fluorescein sodium were acquired from Sigma-Aldrich (St. Louis, MO, USA). Sodium carbonate was obtained from Riedel-de Haën (Seelze, Germany). Finally, potassium ferricyanide, sodium hydrogen phosphate, and sodium dihydrogen phosphate were obtained from Showa Chemical (Tokyo, Japan).

### 3.2. Preparation of RPHs

RPHs were prepared as previously described, except that fungal amylase was adopted to saccharify the starch in rice flour, avoiding damage to amino acids caused by bacterial amylase hydrolysis at high temperatures [43,58]. One hundred grams of rice flour was soaked in 1000 mL of distilled water containing 0.5% fungal amylase (Genencor, NY, USA); the mixture was subsequently heated for 24 h to 60 °C (pH 4.2), after which it was allowed to cool to room temperature. Centrifugation was performed for 10 min at 1968× *g* to remove the remaining supernatant. After 20-fold water and 2 mL of 0.1% hydrolytic protease (Healthmate, Changhua, Taiwan) was added to the insoluble portion, the solution was shaken and incubated for 4 h at 55 °C. The pH-stat method was employed to maintain the solution’s pH at the optimal level, and 85 °C heating was then performed for 10 min for enzyme inactivation. The remaining insoluble fraction was removed through centrifugation for 15 min at 3075× *g*. Lyophilization was performed on the supernatant, which was then stored at −20 °C before use.

### 3.3. Antioxidant Activities of RPHs

#### 3.3.1. Total Phenolic Concentration (TPC)

The Folin–Ciocalteu method for discovering the TPC of RPHs was employed [59]. First, 200 μL of Folin–Ciocalteu’s phenol reagent (0.3M) was uniformly mixed through 5-min shaking with 200 μL of RPH solution, and to this mixture, 400 μL of deionized (DI) water and 200 μL of 10% (*w*/*v*) sodium carbonate solution were added. The mixed solution underwent 60 min of incubation in darkness at room temperature. It was then centrifuged for 15 min at 3000 rpm. The measurement used 100 μL of supernatant. To determine the TPC (unit: mg) of the gallic acid equivalent (GAE) per gram of dry RPH sample (unit: mg GAE/g RPHs), the optical absorbance data were input to a standard curve representing gallic acid. The absorbance was obtained at 700 nm by using Epoch Microplate Spectrophotometer (BioTek, VT, USA).

#### 3.3.2. Total Flavonoid Content (TFC) 

TFC was obtained following the approach of Wathoni et al. with minor modifications [60]. First, 500 μL each of the sample and 2% (*w*/*v*) aluminum chloride solution were mixed. The reaction solution was mixed thoroughly and left for 10 min, and the absorbance at 415 nm was evaluated. The result is reported in micrograms of quercetin equivalent (QE) per gram of the dry RPH sample (µg QE/g RPHs).

#### 3.3.3. DPPH Free Radical Scavenging Activity

First, 198 μM DPPH ethanol solution (50 μL) and the RPH solution or DI water (0.5 μL; the sample and control, respectively) were mixed and then let to stand for 30 min in darkness at room temperature. The solution’s absorbance at 517 nm was subsequently obtained. Relative scavenging activity was computed by determining the absorbance difference between the sample and control. High DPPH free radical scavenging activity was reflected by low optical absorbance. In the RPH solution’s DPPH free radical scavenging activity assessment, the standard employed was vitamin C [61,62,63].

(1) $$\mathrm{DPPH}\;\mathrm{free}\;\mathrm{radical}\;\mathrm{scavenging}\;\mathrm{activity}\;\left(\%\right)=\left[1-\frac{\left(A_{517}\;\mathrm{of}\;\mathrm{the}\;\mathrm{sample}\right)}{\left(A_{517}\;\mathrm{of}\;\mathrm{the}\;\mathrm{control}\right)}\right]\times 100\%$$

#### 3.3.4. Scavenging Activity of ABTS Free Radicals

The approach reported by Wu et al. was employed to evaluate the RPH solution’s antioxidant activity [64]. First, 7 mM ABTS stock solution (250 μL) was reacted with 2.45 mM potassium persulfate (250 μL) to yield the ABTS free radical cation (ABTS^•+^), with the mixture being kept for 16 h at 4 °C in darkness before it was employed. After equilibration in darkness at room temperature, 0.1 M phosphate-buffered saline (PBS; pH 7.4) was employed to dilute the solution to 0.70 ± 0.02 absorbance at 734 nm. Then, to 180 μL of diluted ABTS solution, 20 µL of Trolox (positive control) or the RPH solution (sample) was added. The mixture was then subjected to 10 min of room-temperature incubation. This study determined the optical absorbance at 734 nm; lower absorbance corresponded to higher ABTS free radical scavenging activity. The standard utilized for assessing the RPH solution’s ABTS free radical scavenging activity was the antioxidant Trolox.

(2) $$\mathrm{ABTS}\;\mathrm{free}\;\mathrm{radical}\;\mathrm{scavenging}\;\mathrm{activity}\;\left(\%\right)=\left[1-\frac{\left(A_{734}\;\mathrm{of}\;\mathrm{the}\;\mathrm{sample}\right)}{\left(A_{734}\;\mathrm{of}\;\mathrm{the}\;\mathrm{control}\right)}\right]\times 100\%$$

#### 3.3.5. Reduction Capacity

The ferric-reducing antioxidant power assay was employed to determine the RPH solution’s total antioxidant activity. As reported by Lin et al. [29], the RPH solution (200 μL) was uniformly mixed with 1% (*w*/*v*) K_3_Fe(CN)_6_ and 0.2 M PBS buffer (pH 6.6; 100 μL each). For 20 min, a 50 °C water bath was employed to heat the mixture; after removal of the mixture from the bath, it was quickly cooled for 3 min. Subsequently, an addition of 10% (*w*/*v*) trichloroacetic acid (100 μL) and 10-min centrifugation at 3000 rpm were performed. This was followed by extraction of the supernatant (400 μL) and its uniform mixing with 0.1% (*w*/*v*) FeCl_3_ (100 μL) and DI water (400 μL). Fe_4_[Fe(CN)_6_]_3_ was obtained through 10-min reaction of this mixture in darkness. Subsequently, a higher optical absorbance (measured at 700 nm) indicated higher reduction capacity. The standard vitamin C was utilized to determine the vitamin C equivalent (VCE) content per gram of RPHs.

#### 3.3.6. Oxygen Radical Absorbance Capacity (ORAC)

This study obtained the ORAC by modifying a previously reported method [65]. After dissolution of the RPH sample in distilled water, the RPH solution (50 µL) was mixed with fluorescein (10 µM) in a 96-well microtiter plate. The solution underwent 15-min incubation at 37 °C followed by the addition of 50 µL of AAPH (500 mM). Every 5 min and over a total of 120 min, the fluorescence was recorded (λ_ex_ and λ_em_ = 485 and 528 nm, respectively). The antioxidant capacity of RPHs was discovered from the fluorescence decay kinetics by calculating the area under the curve (AUC). In computing the RPH ORAC, the standard was 15–250 µM Trolox. The ORAC is reported as micromoles of Trolox equivalent (TE) per gram of dry RPH sample (µmol TE/g RPHs).

### 3.4. Hyaluronidase Inhibitory Activity

The hyaluronidase inhibition test was performed using a 96-well microplate and a previously reported method with slight modifications [40]. N-acetylglucosamine was released by reacting hyaluronidase with the HA substrate. In the presence of any inhibitor, the N-acetylglucosamine release was reduced, with this release detecting by obtaining the 600-nm absorbance. HA was precipitated with acidic albumin solution composed of 0.1 M acetate buffer (pH 3.9) and bovine serum albumin (1 mg/mL). The sample solution and 5 mg/mL hyaluronidase underwent 20-min incubation at 37 °C. To the incubation mixture, HA (100 μL; 5.0 mg/mL in 0.1 M acetate buffer) was subsequently added. Further incubation at 37 °C for 40 min was performed. 0.1 mL 0.4 M alkaline borate solution was added to halt the enzymatic reaction.

### 3.5. Tyrosinase-Inhibitory Activity

The present study evaluated the antityrosinase activity of RPHs by using a previously reported protocol with modifications [66]. An enzyme solution (135 U/mL) was prepared by dissolving tyrosinase in 20 mM phosphate buffer (pH 6.8). Additionally, DI water was employed for 1.25 mM L-DOPA solution preparation. Then, 40 μL of various concentrations of RPH sample solutions were mixed with 40 μL of tyrosinase solution and 120 μL of L-DOPA solution. For 30 min, this mixture was kept at 37 °C in the test of RPHs’ inhibition of tyrosinase activity. A spectrophotometer (FLUOstar Omega Microplate Reader, BMG Labtech GmbH, Germany) was employed to obtain the 475-nm absorbance. All measurements were performed three times. The absorbance of the corresponding group when tyrosinase was not present was subtracted. The enzyme inhibition rate was determined as

(3) $$\mathrm{Tyrosinase}\;\mathrm{inhibition}\;\left(\%\right)=\left[1-\frac{\left(A_{475}\;\mathrm{of}\;\mathrm{the}\;\mathrm{sample}\right)}{\left(A_{475}\;\mathrm{of}\;\mathrm{the}\;\mathrm{control}\right)}\right]\times 100\%$$

### 3.6. Characterization of RPHs

#### 3.6.1. Amino Acid Profiles

This study discovered the amino acid composition of RPHs. First, for 24 h and at 115 °C, 4 M methanesulfonic acid was employed to hydrolyze the samples in evacuated sealed tubes. Two Waters 510 solvent delivery systems and an amino acid analyzer (L-8900; Hitachi, Tokyo, Japan) were employed for derivatized amino acid separation on a Spherisorb ODS2 column measuring 25 m × 64.6 mm. This study employed the following solvents: (a) sodium acetate (0.14 M) and triethylamine (850 µL/L; pH 5.6) and (b) 60% acetonitrile, for which the gradient was 0% for 2 min; 0–42% for 15.5 min (convex curve); and 100% for 4 min. Duplicate samples were taken for the measurement of amino acid profiles at 254 nm [67,68].

#### 3.6.2. Molecular Weight (MW) of Protein

In accordance with Schägger’s method [69] and under reducing conditions, this study obtained the MW distribution through tricine–sodium dodecyl sulfate (SDS)–polyacrylamide gel electrophoresis (PAGE) with slight modifications. A sample buffer (30 g/L SDS, 0.375 M Tris-HCl, 0.125 g/L Coomassie Brilliant Blue G-250, and 75 g/L glycerol; pH 7) was employed to disperse the freeze-dried sample, with centrifugation then performed before loading. A total of 20 µL 2-mercaptoethanol was added to 1 mL of the tricine-SDS-PAGE sample. The sample was heated at 100 °C for 90 s. A sample well was loaded with each sample and Unstained Protein Standard Broad Range (Bio-Rad Laboratories, Germany) by using a microsyringe. Electrophoresis was then performed—first at a constant 30 mV until the entire sample was contained within the stacking gel and afterwards until completion at a constant 100 mV. Subsequently, 0.02% Coomassie Brilliant Blue R-250 solution was applied for gel staining. Absolute background destaining of the gels was performed by shaking the gels in 10% acetic acid overnight. Finally, the gel image was analyzed to identify the protein bands in the lanes; this analysis was performed in ImageJ (U.S. National Institutes of Health, Bethesda, MD, USA). Standard markers were employed to obtain a calibration curve from which the MW was estimated. Briefly, the first step was to determine each band’s length of migration (Rf) from the top of the separating gel. The second step was the calculation of the calibration curve by employing Rf and log (MW) for a standard marker with a given MW. MW determination was performed using the Rf of protein bands in RPHs.

### 3.7. Cytotoxicity Assay

Raw 264.7 cells were cultured in high glucose Dulbecco’s Modified Eagle Medium (DMEM) containing 10% fetal bovine serum (FBS), 4.5 g/L Glucose, 1% antibiotic solution (100 units/mL penicillin and 100 μg/mL Streptomycin), 4 mM L-Glutamin and 1.5 g/L sodium bicarbonate at 37 °C and 5% CO_2_. The cell toxicity of raw 264.7 cells for RPHs was measured by a 3-(4,5-dimethylthiazol-2-yl)-2,5 diphenyl-tetrazolium bromide (MTT) proliferation assay method. About 1 × 10^4^ cells per well were plated in 96-well plates. After 24 h, various concentrations of RPHs (0–2000 μg/mL) were added into the cells. After 24 and 48 h of incubation, 100 µL of MTT solution (0.5 mg/mL) was added. Blue formazan crystals were observed when checked under a microscope. DMEM was removed and 100 µL of dimethyl sulfoxide (DMSO) was added per well. The absorbance was measured using a microtiter plate reader. The cell viability (%) was then calculated as [A_570_ (treated cells) − A_570_ (background)] / [A_570_ (untreated cells) − A_570_ (background)] × 100% [70].

### 3.8. Statistical Analysis

The report for each hydrolysate sample was the average value from three independent repeated experiments and determinations. Results expressed in mean ± standard deviation (S.D.) were analyzed by one-way ANOVA and Duncan’s post hoc test using the Statistical Analysis System (version 20.0; SPSS, Armonk, NY, USA). Values of *p* < 0.05 were considered to be statistical significance. 

## 4. Conclusions

This study examined the functions of RPHs. Experimental results revealed that RPHs contained phenolic compounds and flavonoids and exhibited a range of antioxidant activities, such as DPPH and ABTS scavenging activities, reduction capacity, and ORAC. In addition, RPHs effectively inhibited tyrosinase and hyaluronidase activities. The protease was a critical factor affecting the MW patterns of RPHs. The analysis of RPHs indicates their potential for use as an ingredient in cosmetics.

## Figures and Tables

**Table 1:** Amino acid profiles of rice protein hydrolysate (RPH) samples.

Amino Acid Profiles	Quantity in RPH (g/100 g) ^#^
Alanine	0.37
Arginine	0.41
Aspartic acid	0.52
Cystine	0.09
Glutamic acid	0.85
Glycine	0.27
Histidine	0.12
Isoleucine	0.23
Leucine	0.43
Lysine	0.25
Methionine	0.04
Phenylalanine	0.26
Proline	0.24
Serine	0.29
Threonine	0.21
Tryptophan	-
Tyrosine	0.27
Valine	0.32
Total amino acids (TAA)	5.18
Essential amino acids (EAA)	1.70
Branched chain amino acids (BCAA)	0.62

^#^ Values are the mean of duplicate measurements.

## Data Availability

The data presented in this study are available on request from the corresponding author.

## Sample Availability

Not available.
